# Development and Internal Validation of Supervised Machine Learning Algorithm for Predicting the Risk of Recollapse Following Minimally Invasive Kyphoplasty in Osteoporotic Vertebral Compression Fractures

**DOI:** 10.3389/fpubh.2022.874672

**Published:** 2022-05-02

**Authors:** Sheng-tao Dong, Jieyang Zhu, Hua Yang, Guangyi Huang, Chenning Zhao, Bo Yuan

**Affiliations:** ^1^Department of Spine Surgery, Second Affiliated Hospital of Dalian Medical University, Dalian, China; ^2^Department of Otolaryngology, Head and Neck Surgery, Second Affiliated Hospital of Dalian Medical University, Dalian, China; ^3^Department of Orthopedics, Second Affiliated Hospital of Dalian Medical University, Dalian, China; ^4^Department of Reparative and Reconstructive Surgery, Second Affiliated Hospital of Dalian Medical University, Dalian, China

**Keywords:** support vector machine, recollapse, osteoporotic vertebral compression fractures, percutaneous kyphoplasty, risk factors

## Abstract

**Background:**

The published literatures indicate that patients with osteoporotic vertebral compression fractures (OVCFs) benefit significantly from percutaneous kyphoplasty (PKP), but this surgical technique is associated with frequent postoperative recollapse, a complication that severely limits long-term postoperative functional recovery.

**Methods:**

This study retrospectively analyzed single-segment OVCF patients who underwent bilateral PKP at our academic center from January 1, 2017 to September 30, 2019. Comparing the plain films of patients within 3 days after surgery and at the final follow-up, we classified patients with more than 10% loss of sagittal anterior height as the recollapse group. Univariate and multivariate logistic regression analyses were performed to determine the risk factors affecting recollapse after PKP. Based on the logistic regression results, we constructed one support vector machine (SVM) classifier to predict recollapse using machine learning (ML) algorithm. The predictive performance of this prediction model was validated by the receiver operating characteristic (ROC) curve, 10-fold cross validation, and confusion matrix.

**Results:**

Among the 346 consecutive patients (346 vertebral bodies in total), postoperative recollapse was observed in 40 patients (11.56%). The results of the multivariate logistical regression analysis showed that high body mass index (BMI) (Odds ratio [OR]: 2.08, 95% confidence interval [CI]: 1.58–2.72, *p* < 0.001), low bone mineral density (BMD) T-scores (OR: 4.27, 95% CI: 1.55–11.75, *p* = 0.005), presence of intravertebral vacuum cleft (IVC) (OR: 3.10, 95% CI: 1.21–7.99, *p* = 0.019), separated cement masses (OR: 3.10, 95% CI: 1.21–7.99, *p* = 0.019), cranial endplate or anterior cortical wall violation (OR: 0.17, 95% CI: 0.04–0.79, *p* = 0.024), cement-contacted upper endplate alone (OR: 4.39, 95% CI: 1.20–16.08, *p* = 0.025), and thoracolumbar fracture (OR: 6.17, 95% CI: 1.04–36.71, *p* = 0.045) were identified as independent risk factors for recollapse after a kyphoplasty surgery. Furthermore, the evaluation indices demonstrated a superior predictive performance of the constructed SVM model, including mean area under receiver operating characteristic curve (AUC) of 0.81, maximum AUC of 0.85, accuracy of 0.81, precision of 0.89, and sensitivity of 0.98.

**Conclusions:**

For patients with OVCFs, the risk factors leading to postoperative recollapse were multidimensional. The predictive model we constructed provided insights into treatment strategies targeting secondary recollapse prevention.

## Introduction

Osteoporotic vertebral compression fractures (OVCFs) have become a frequent low-energy injury with the accelerated aging of the population worldwide (1, 2). Approximately 1.4 million patients worldwide are diagnosed with fresh OVCF each year (3). Patients suffer from chronic back pain, reduced mobility and daily activities, poor quality of life, and the accompanying heavy financial burden. Percutaneous kyphoplasty (PKP) is a minimally invasive surgical option widely performed to treat symptomatic OVCFs as it provides rapid pain relief, restoration of local kyphotic deformity, and early mobilization (4–6). It is worth noting that the augmented vertebral body recollapse is a common and serious complication after PKP technique, and deserves extensive caution as it is regularly the primary culprit for low back pain and functional impairment (1, 2, 7). The published literatures suggest less cement volume, sarcopenia, low bone mineral density (BMD), advanced age, and being female as risk factors for recollapse, but comprehensive and innovative risk factor selection criteria and predictive models are absent (2, 8–10).

The well-known features of machine learning (ML), which is affiliated with artificial intelligence (AI), for automatic learning and improvement of complex relationships are developed based on the recognition of patterns. ML algorithms have made a profound contribution to solve intractable problems in the medical field (11–13). As one of the classical ML algorithms, support vector machines (SVMs) exhibit excellent classification capabilities and play an irreplaceable role in the osteoporosis–AI intersection (14, 15). Given the remarkable contribution of ML algorithms in the field of medical big data, this work attempts to develop a predictive model for assessing recollapse based on routine clinical data.

The purpose of this study was to investigate the rate of recollapse in patients with OVCFs after PKP. Furthermore, the SVM model was constructed to explore the predictors significantly associated with augmented vertebral recollapse.

## Methods

### Study Design and Participants

The study retrospectively analyzed patients with single-segment OVCFs treated with bilateral PKP at our hospital from January 1, 2017 to September 30, 2019. Targeted patients were selected according to specific inclusion and exclusion criteria.

The inclusion criteria were as follows: (1) the patients' diagnoses of OVCF were supported by radiological evidence (a hypointense signal on T1-weighted images and a hyperintense signal on T2-weighted images of MRI, T-scores < −1.5 of dual-energy X-ray); (2) acute or subacute symptomatic OVCFs without neurological deficits; (3) no serious surgical contraindications and have undergone single-segment PKP; (4) available general information, radiological outcomes, and operative records; (5) age ranging from 50 to 80 years; (6) at least 1 year postoperative follow-up. The exclusion criteria were as follows: (1) patients rated as III or higher in the American Society of Anesthesiologists (ASA) classification; (2) patients have undergone spinal surgery previously; (3) patients diagnosed with spinal malignancies, pathological fractures, deformities, infections, or other spinal disorders; (4) patients had bone cement allergy or developed severe postoperative complications, including pulmonary cement embolism and deep vein thrombosis. All patients were evaluated at 1 week and then 1, 3, 6 months, and 1 year postoperatively.

The approval was obtained from the ethics committee of our institution, and patients' data were collected from the medical electronic system and telephone surveys.

### Data Collection

General information, including age, gender, BMI, BMD, diabetes mellitus, smoking status, period of surgery to recollapse, and preoperative and postoperative visual analog scale (VAS, 0–10), were obtained from our medical records. Operative records included treated level, anesthesia methods, and cement volume.

**Figure 1:**
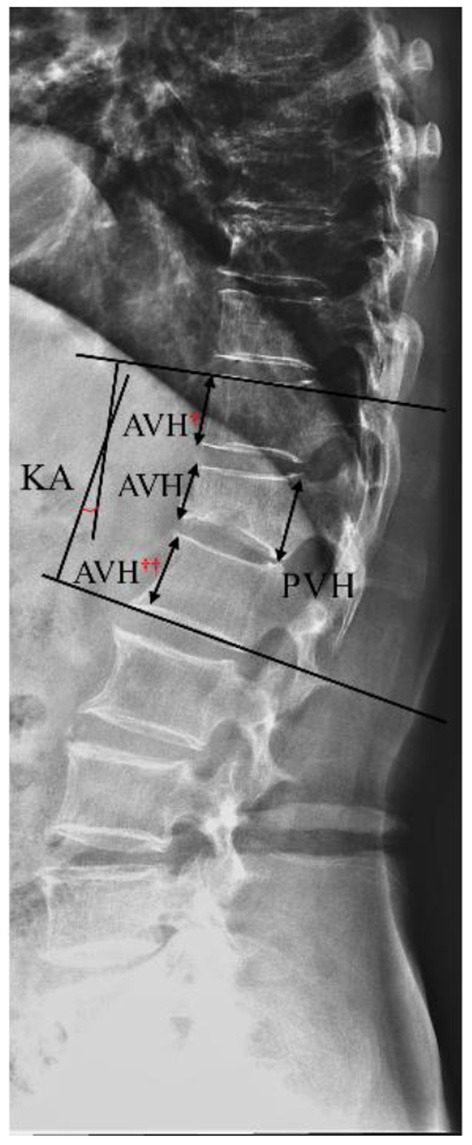
Schematic diagram of vertebral height loss and local kyphotic angle (KA). Vertebral body compression rate (VBCR) = anterior vertebral height (AVH)/posterior vertebral height (PVH) × 100%; percentage of anterior height compression (PAHC) = AVH/[(AVH^***†***^ + AVH^***††***^)/2] × 100%.

**Figure 2:**
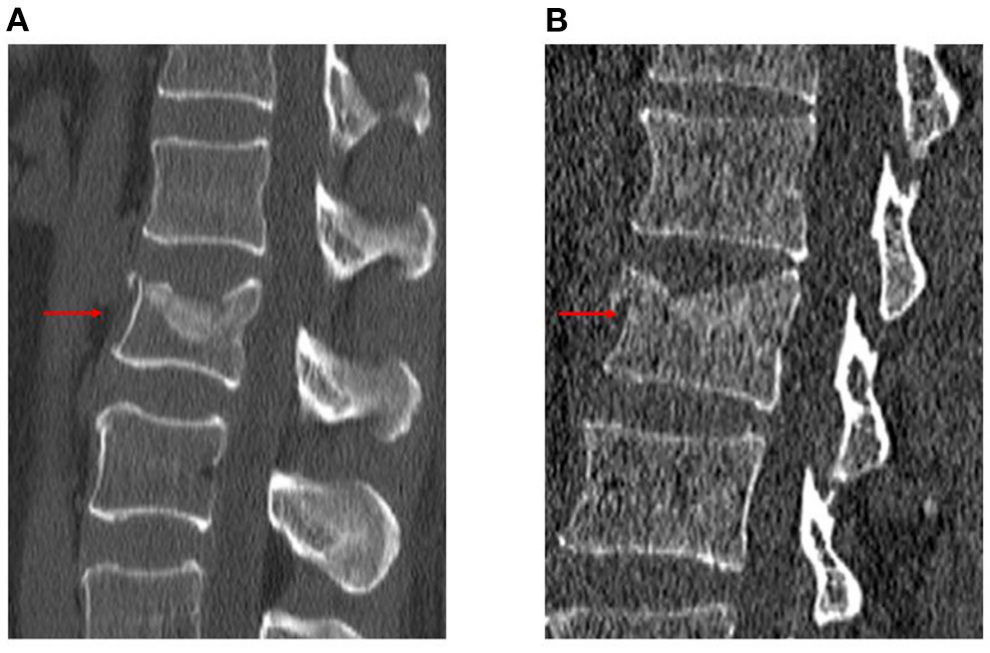
Schematic diagram of fracture localization. Cranial endplate fracture **(A)**. Cranial endplate and anterior cortical wall fracture **(B)**.

**Figure 3:**
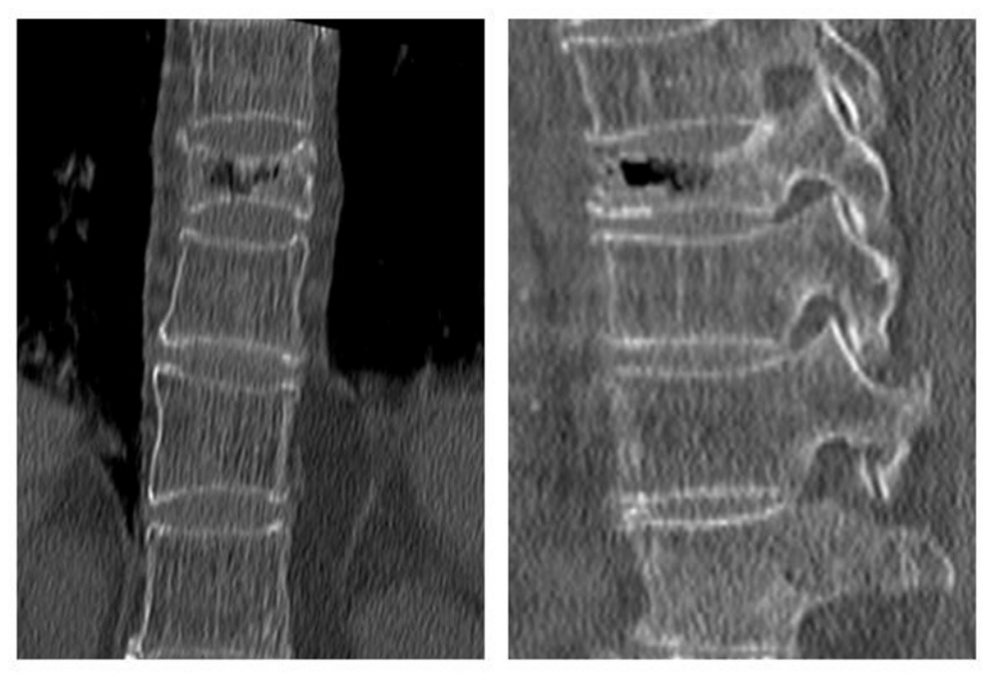
Intravertebral vacuum cleft (IVC) was demonstrated by computed tomography at L1 level.

**Figure 4:**
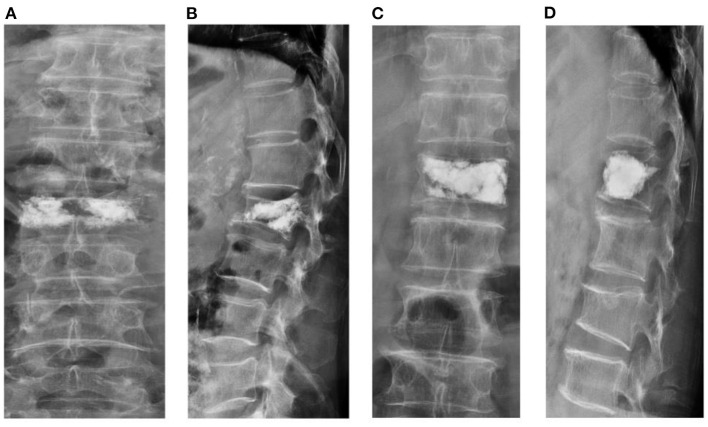
Schematic diagram of vertebral body height restoration after percutaneous kyphoplasty (PKP). Separated cement mass **(A,B)**. Cement-contacted upper endplate with integrated cement mass **(C,D)**.

**Figure 5:**
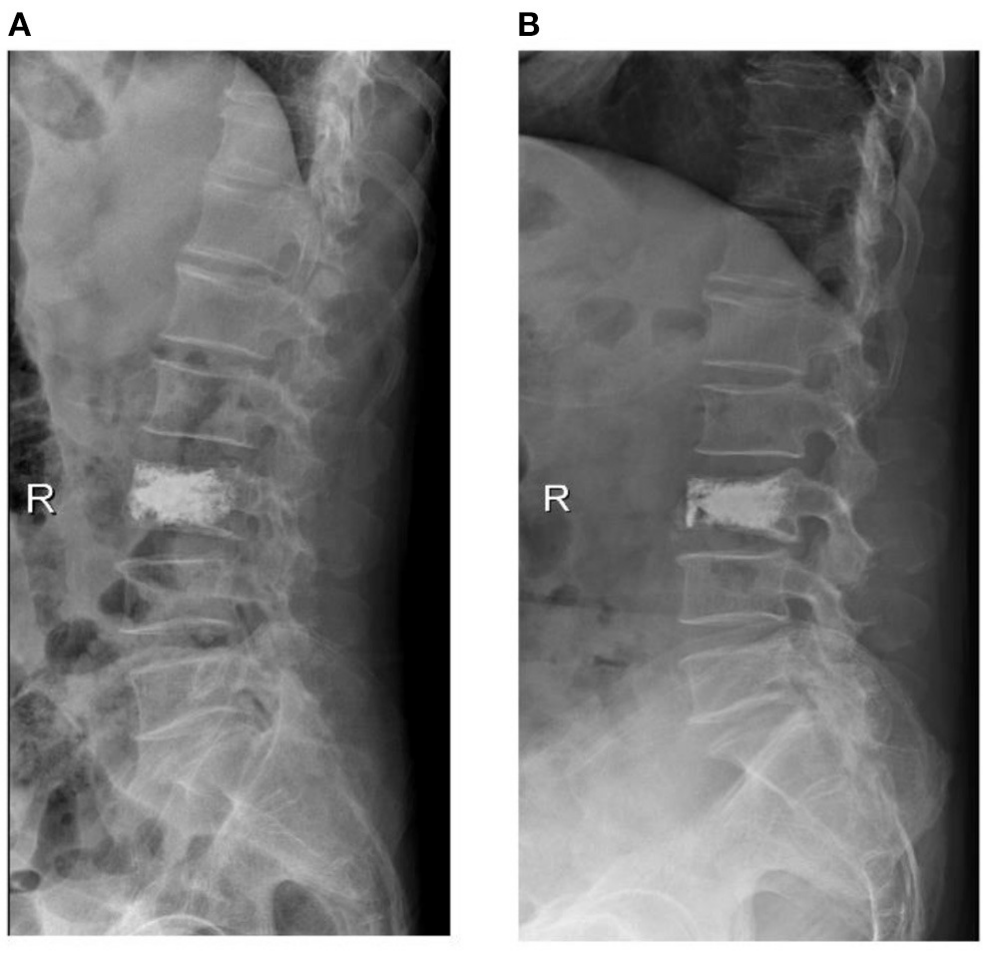
X-ray of a 65-year-old male with osteoporotic vertebral compression fracture (L3), he was referred to our hospital at 10 months postoperatively for low back pain and was diagnosed with recollapse.

**Figure 6:**
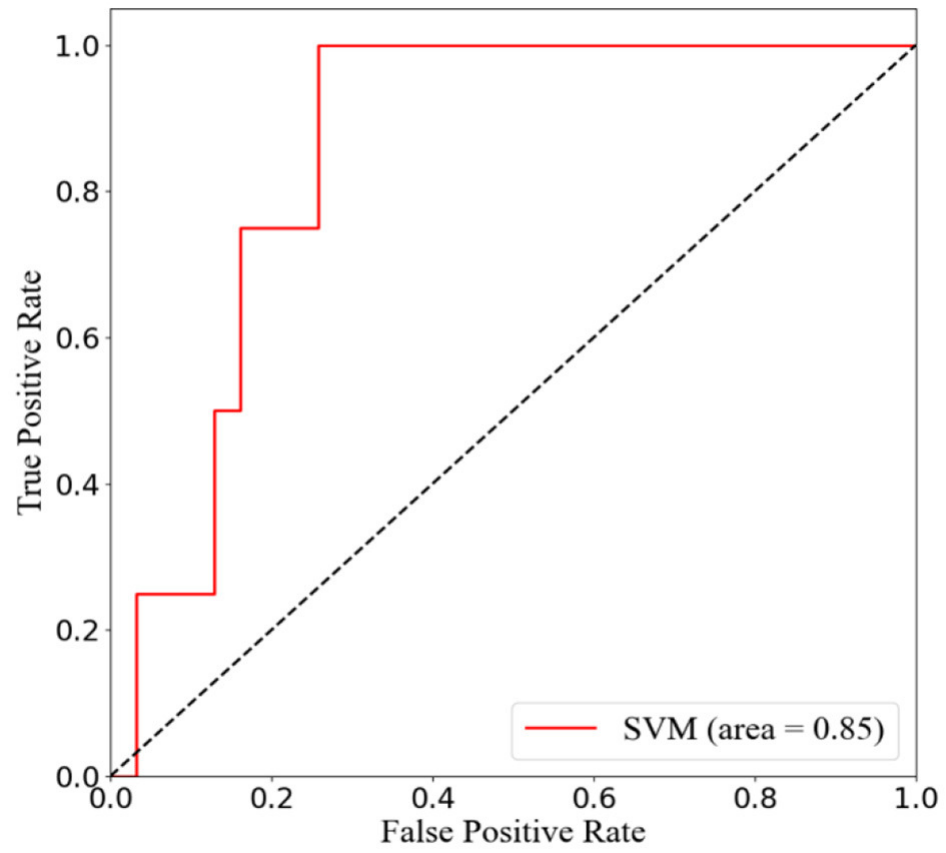
Receiver operation characteristic (ROC) curve analysis of support vector machine (SVM) model with the maximum value 0.85.

The following radiological outcomes were measured and compared: (1) vertebral body compression rate (VBCR), which is a ratio of anterior vertebral height (AVH) to posterior vertebral height (PVH); (2) percentage of anterior height compression (PAHC), which is a ratio of the AVH between the fractured and the superior and inferior adjacent segments; (3) local kyphotic angle, which is the angle between the upper and lower endplates of the fractured segment (Figure 1); (4) fracture localization, including cranial endplate fractures, anterior cortical wall fractures, and both without violation (Figure 2); (5) intravertebral vacuum cleft (IVC) (Figure 3); (6) vertebral height recovery, which is the difference between postoperative and preoperative VBCR and PAHC; (7) local kyphotic recovery, which is the difference between preoperative and postoperative local kyphotic angle; (8) cement leakage; (9) cement distribution, including separated and integrated cement masses (16); (10) cement-contacted endplates, defined as both upper and lower endplates in contact with bone cement (Figure 4). Furthermore, all the radiological evaluations were completed by two experienced spine surgeons.

We defined the recollapsed group based on whether sagittal anterior vertebral height is lost more than 10% compared to postoperative radiographs during follow-up (Figure 5). In addition, all cases were recommended a standard anti-osteoporosis treatment (calcium, vitamin D, and diphosphate) at 1 year follow-up.

### Support Vector Machine

The SVM algorithm demonstrates good classification performance by transforming the input space into a high-dimensional space using a nonlinear function called a kernel function. Data in the format “. xlxs” were provided for the SVM algorithm, the data-driven automatic modeling mechanism operated with the assistance of two parameters, the error penalty parameter C and the γ coefficient. The receiver operating characteristic (ROC) curve and 10-fold cross-validation were used to evaluate the predictive performance of SVM classifier. The confusion matrix showed the classification results. All data analyses were performed in the Python programming language (Python 3.8.0, https://www.python.org/). These indices provided evidence for performance evaluation and presented the equations as follows:


$$\begin{array}{l} Accuracy\;\left(ACC\right)=\frac{TP+TN}{TP+TN+FP+FN}\\ \;Precision=\frac{TP}{TP+FP}\;\\ \;Sensitivity=\frac{TP}{TP+FN}\; \end{array}$$

### Statistical Analysis

All statistical analyses were conducted using SPSS version 22.0 (IBM SPSS, Armonk, New York). Continuous variables were compared with Student's *t-*tests and presented as means ± standard deviations (SD), and categorical variables that were presented as relative frequencies and percentages were examined by the Chi-square test. In addition, the variables with a statistical difference in the univariate analysis were input into multivariate regression to investigate the independent risk factors. Furthermore, these significant indexes were provided to the automatically learned SVM model. Satisfactory statistical significance was defined as *p* < 0.05.

## Results

### Baseline Clinical Characteristics

Among the 346 consecutive patients (346 vertebral bodies in total) who underwent PKP successfully at our department, 40 cases (11.56%) were observed with more than 10% of vertebral height loss and were defined as the recollapsed group during 1 year follow-up. As shown in Tables 1, 2, the differences in demographic characteristics (age, sex, VAS, diabetes mellitus, and smoking status), anesthesia type, cement leakage, and radiological indices (VBCR, PAHC, and cement-contacted endplates) between recollapsed group and non-recollapsed group were not statistically significant (*p* > 0.05). Furthermore, compared with non-recollapsed group, the BMI, BMD, incidence of thoracolumbar fractures (T11–L2) and IVC, recovered local kyphotic angle, and frequency of integrated cement masses were significantly higher, and cement volume, incidence of cranial endplate or anterior cortical wall intact were significantly lower in recollapsed patients. Previous literature has reported smoking as a risk factor for progressive kyphotic deformity, but we could not confirm the relationship between smoking and recollapse, presumably due to the gender-specific nature of the smoking population in the Chinese social environment. The majority of the population with OVCFs is female but they rarely smoke (8.75 vs. 22.64%). In addition, postoperative patient-reported outcomes (PROs) indicated comparable surgical benefits regardless of whether the patient subsequently recollapsed. In the series of high-risk patients, the average period of surgery to recollapse was 8.4 months.

**Table 1:** Descriptive summary of patient cohort.

**Variable**	**Total (*n* = 346)**	**Recollapsed group (*n* = 40)**	**Non-recollapsed group (*n* = 306)**	***p***
Age (years)	64.11 ± 8.48	64.85 ± 7.98	64.01 ± 8.54	0.559
Sex (%)				0.524
Female	240 (69.36)	26 (65.00)	214 (69.94)	
Male	106 (30.64)	14 (35.00)	92 (30.06)	
BMI (kg/ m^2^)	23.64 ± 1.99	25.71 ± 1.29	23.37 ± 1.90	<0.001
BMD (T score)	−3.27 ± 0.52	−3.48 ± 0.42	−3.24 ± 0.53	0.002
Preoperative VAS	6.05 ± 0.82	6.08 ± 0.79	6.05 ± 0.82	0.851
Postoperative VAS	2.41 ± 1.08	2.40 ± 1.16	2.41 ± 1.07	0.949
Diabetes mellitus (%)				0.996
Yes	52 (15.03)	6 (15.00)	46 (15.03)	
No	294 (84.97)	34 (85.00)	260 (84.97)	
Smoking status (%)				0.919
Yes	45 (13.01)	5 (12.50)	40 (13.07)	
No	301 (86.99)	35 (87.50%)	266 (86.93)	
Treated level (%)				0.001
T4–T10	68 (19.65)	2 (5.00)	66 (21.57)	
T11–L2	146 (42.20)	27 (67.50)	119 (38.89)	
L3–L5	132 (38.15)	11 (27.50)	121 (39.54)	
Anesthesia (%)				0.941
Local	284 (82.08)	33 (82.50)	251 (82.03)	
General	62 (17.92)	7 (17.50)	55 (17.97)	
Cement volume (ml)	3.37 ± 0.57	3.09 ± 0.53	3.40 ± 0.56	<0.001
Cement leakage (%)				0.498
Yes	33 (9.54)	5 (12.50)	28 (9.15)	
No	313 (90.46)	35 (87.50)	278 (90.85)	
Period of surgery to recollapse (months)	8.40 ± 1.74			

*Data are displayed as mean ± SD, number (%), BMI, body mass index; BMD, bone mineral density; VAS, Visual analog scores*.

**Table 2:** Comparison of the radiologic indeces between patients with and without recollapse.

**Variable**	**Total (*n =* 346)**	**Recollapsed group (*n =* 40)**	**Non-recollapsed group (*n =* 306)**	***p***
**VBCR (%)**				
Preop	67.98 ± 2.29	68.14 ± 2.34	67.96 ± 2.28	0.647
Postop	91.11 ± 3.27	90.57 ± 3.04	91.18 ± 3.30	0.265
Recovery	23.12 ± 3.86	22.43 ± 3.26	23.22 ± 3.92	0.224
**PAHC (%)**				
Preop	67.88 ± 2.90	67.81 ± 2.72	67.89 ± 2.92	0.881
Postop	89.94 ± 4.92	89.79 ± 5.31	89.96 ± 4.87	0.840
Recovery	22.06 ± 5.77	21.98 ± 5.98	22.07 ± 5.74	0.922
**Local kyphotic angle (°)**				
Preop	21.56 ± 2.07	21.79 ± 1.98	21.54 ± 2.08	0.471
Postop	14.29 ± 2.01	13.48 ± 1.77	14.40 ± 2.01	0.006
Recovery	7.32 ± 2.92	8.86 ± 2.52	7.11 ± 2.91	<0.001
Fracture localization (%)				<0.001
Cranial endplate fractures	91 (26.30)	15 (37.50)	76 (24.84)	
Anterior cortical wall fractures	73 (21.10)	11 (27.50)	62 (20.26)	
Both violation	57 (16.47)	11 (27.50)	46 (15.03)	
Both intact	125 (36.13)	3 (7.50)	122 (39.87)	
IVC (%)				0.020
Yes	28 (8.09)	7 (17.50)	21 (6.86)	
No	318 (91.91)	33 (82.50)	285 (93.14)	
Cement distribution (%)				<0.001
Separated	103 (29.77)	21 (52.50)	82 (26.80)	
Integrated	243 (70.23)	19 (47.50)	224 (73.20)	
Cement-contacted endplates (%)				0.056
Upper endplate alone	81 (23.41)	13 (32.50)	68 (22.22)	
Lower endplate alone	91 (26.30)	14 (35.00)	77 (25.16)	
Both contacted	116 (33.53)	6 (15.00)	110 (35.95)	
None contacted	58 (16.76)	7 (17.50)	51 (16.67)	

*Preop, preoperative; Postop, postoperative; VBCR, vertebral body compression rate; PAHC, percentage of anterior height compression; IVC, intravertebral vacuum cleft*.

### Multivariate Analysis for Risk Factors of Recollapse

The results of the univariate analysis showed statistically significant differences between BMI, BMD, IVC, cement volume, postoperative kyphotic angle, recovered kyphotic angle, cement distribution, fractire localization, cement-contacted endplates and treated level by comparing routine variables. The results were then subsequently input to a multivariate logistic regression analysis to investigate the independent risk factors for recollapse. The eventual logistic regression results are presented in Table 3. High BMI (OR: 2.08, 95% CI: 1.58–2.72, *p* < 0.001), low BMD T-scores (OR: 4.27, 95% CI: 1.55–11.75, *p* = 0.005), presence of IVC (OR: 3.10, 95% CI: 1.21–7.99, *p* = 0.019), separated cement distribution (OR: 3.10, 95% CI: 1.21–7.99, *p* = 0.019), cranial endplate or anterior cortical wall violation (OR: 0.17, 95% CI: 0.04–0.79, *p* = 0.024), cement-contacted upper endplate alone (OR: 4.39, 95% CI: 1.20–16.08, *p* = 0.025), and thoracolumbar fracture (OR: 6.17, 95% CI: 1.04–36.71, *p* = 0.045) were identified as independent risk factors for recollapse after a PKP surgery.

**Table 3:** Univariate and multivariate logistic regression analysis for the risk factors of postoperative recollapse after percutaneous kyphoplasty (PKP).

**Variables**	**Univariable logistic regression analysis**				**Multivariable logistic regression analysis**			
	**OR**	**95% CI**		***p***	**OR**	**95% CI**		***p***
		**Lower**	**Upper**			**Lower**	**Upper**	
BMI	1.89	1.54	2.32	<0.001	2.08	1.58	2.72	<0.001
BMD	2.41	1.26	4.60	0.008	4.27	1.55	11.75	0.005
IVC	2.88	1.14	7.28	0.026	3.10	1.21	7.99	0.019
Cement volume	0.36	0.19	0.67	0.001	0.44	0.18	1.07	0.070
Postoperative kyphotic angle	0.79	0.66	0.94	0.007	0.76	0.54	1.07	0.109
Recovered kyphotic angle	1.24	1.10	1.40	0.001	1.10	0.89	1.36	0.396
Cement distribution	3.02	1.55	5.90	0.001	3.10	1.21	7.99	0.019
Fracture localization				0.007				0.066
Cranial endplate fractures	Ref	Ref	Ref	Ref	Ref	Ref	Ref	Ref
Anterior cortical wall fractures	0.90	0.39	2.10	0.805	1.08	0.33	3.46	0.904
Both violation	1.21	0.51	2.86	0.662	1.38	0.39	4.86	0.622
None violation	0.13	0.04	0.45	0.001	0.17	0.04	0.79	0.024
Cement-contacted endplates				0.025				0.037
Both contacted	Ref	Ref	Ref	Ref	Ref	Ref	Ref	Ref
Upper endplate alone	3.51	1.27	9.66	0.015	4.39	1.20	16.08	0.025
Lower endplate alone	3.33	1.23	9.06	0.018	3.86	0.96	15.44	0.056
None contacted	2.52	0.81	7.87	0.113	2.68	0.60	11.99	0.197
Treated level				0.004				0.019
T4–T10	Ref	Ref	Ref	Ref	Ref	Ref	Ref	Ref
T11–L2	7.49	1.73	32.48	0.007	6.17	1.04	36.71	0.045
L3–L5	3.00	0.65	13.94	0.161	1.52	0.24	9.63	0.658

*OR, odds ratio; 95% CI, 95% confidence interval*.

### Development and Validation of Predictive Model

**Figure 7:**
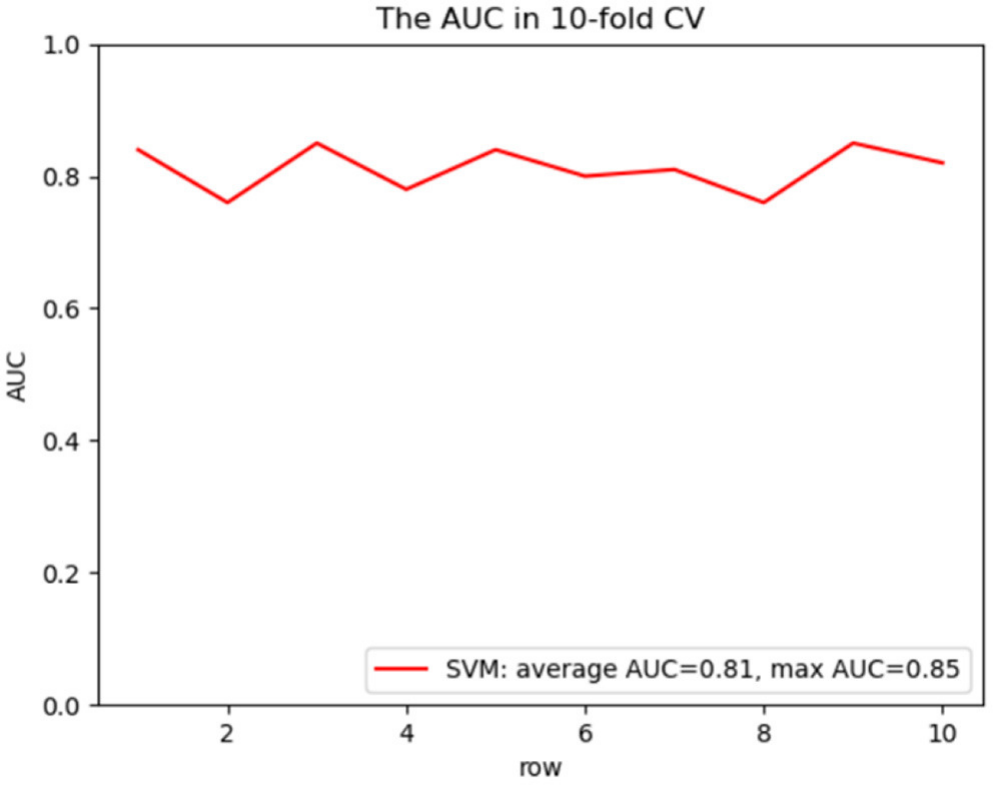
ROC curve analysis of a 10-fold cross validation of SVM model for predicting the risk of recollapse following PKP with average AUC 0.81 and maximum AUC 0.85.

**Figure 8:**
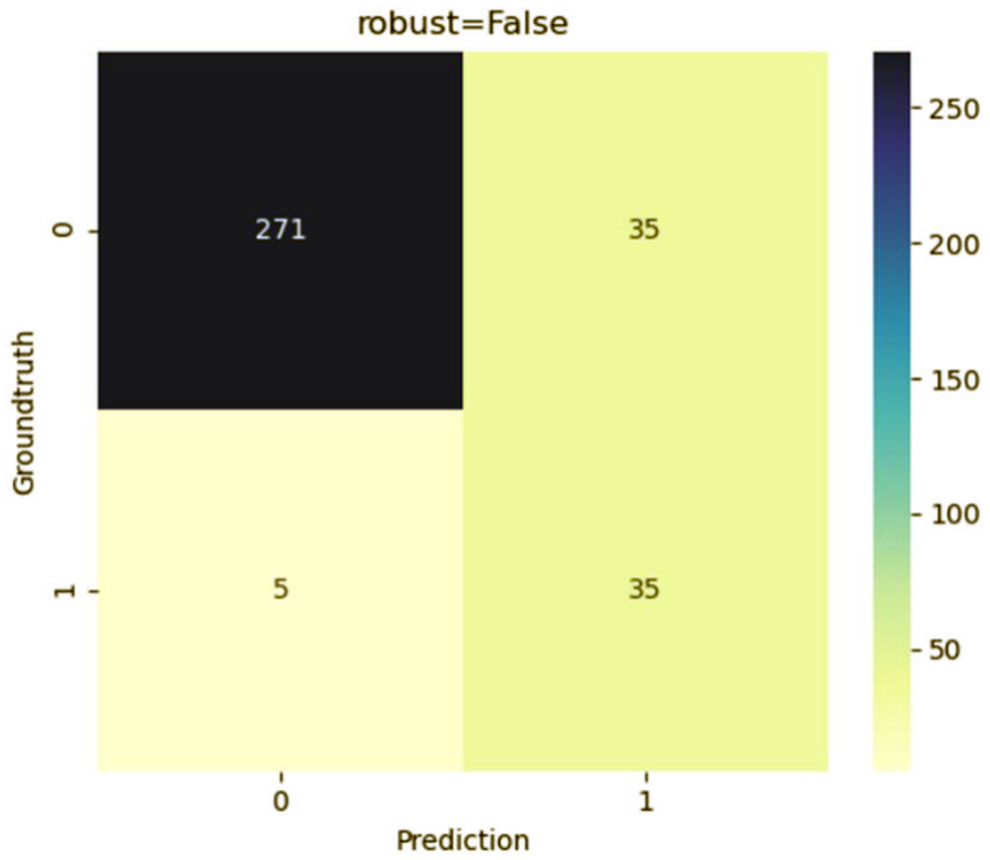
Confusion matrix of SVM model.

To predict the risk of recollapse in patients with OVCFs, we established a predictive model including the seven independent risk factors using the SVM method. As shown in the ROC curve of the prediction model, the area under ROC curve (AUC) was 0.85 (Figure 6). Concurrently, we additionally evaluated the predictive ability of the SVM model using 10-fold cross-validation. Moreover, the following satisfactory evaluation indicators: mean AUC (0.81), maximum AUC (0.85), and accuracy (ACC) (0.88) were obtained, suggesting that the model was excellent at predicting the risk of recollapse after PKP in patients with OVCFs (Figure 7). The confusion matrix further showed the classification results of the algorithm with the precision of 0.89 and sensitivity of 0.98 (Figure 8).

## Discussion

In this study, we developed and validated an SVM model to identify the risk factors significantly associated with recollapse from routine parameters. Numerous studies have shown that ML offers many contributions to accelerate the development of biomedicine (17–19). Particularly in dealing with spinal disorders, SVM models have been applied as decision tools to provide credible evidence for patient-centered treatment strategies (20–22). Although PKP has been considered as a valuable surgical intervention for rapid symptomatic improvement and kyphotic deformity correction (4–6), a recollapse rate of 11.56% at 1 year postoperatively suggests that it is still a hazardous complication. Compared with percutaneous vertebroplasty, the PKP technique suffers from balloon dilatation, which happens to be the most attractive procedure for improving sagittal balance, resulting in impaired cancellous bone structure of the operated vertebrae and an increased recollapse rate (23). Li et al. reported a recollapse rate of ~26% after PKP, and this relatively higher incidence may be explained by the rigorous inclusion criteria and endpoint events in our work (2). To our knowledge, this was the first interdisciplinary study involving ML algorithms to report risk factors for recollapse of OVCFs after PKP.

Severe osteoporosis and obesity increased the risk of recollapse. In our study, low BMD T-scores and high BMI were found to be significant predictors of distinct recollapse and were similar to published conclusions (2, 8, 24–26). Systematically reviewing OVCFs treated with minimally invasive surgery, Klazen et al. identified the critical role of BMD in recollapse (27). Besides, in a 5-year follow-up retrospective study, Lin et al. reported that patients with OVCFs were exposed to a three-fold incidence of refracture due to a disproportionately low BMD (28). The BMD T-scores measured by dual-energy X-ray absorptiometry reflected the bone strength and fracture resistance. The trabecular bone in osteoporotic vertebrae was fragile and vulnerable to reoccurrence of vertebral height loss in response to minor axial loading forces. For patients diagnosed with OVCFs, standard anti-osteoporotic treatment was required postoperatively, but the degree of osteoporosis was still found to be an unignorable trigger for surgical vertebral recollapse in clinical practice. In our study, BMD was lower in the recollapsed group (−3.48 vs. 3.24), which was consistent with other findings. In summary, PKP is effective in restoring vertebral height but fails to prevent subsequent recollapse of the surgical vertebrae due to severe osteoporosis. However, a potential contributor to BMD is obesity, and there was a positive association between BMI and BMD (25). The BMD measured in obese patients from the femoral neck and lumbar spine reflects an exaggerated bone strength due to abdominal thickness and beam sclerosis effects (29). Furthermore, biochemical markers of bone turnover provide insight into the effects of obesity on bone. Leptin produced by subcutaneous adipocytes may inhibit bone formation by increasing sympathetic nervous system activation (30), and secretion of pro-inflammatory cytokines such as interleukin 6 (IL-6) and tumor necrosis factor alpha (TNF-α) by visceral fat increases bone resorption (31). Also, serum 25-hydroxyvitamin D (25OHD) was lower in the obese than in normal weight cohort (25). However, biomechanical analysis of overweight patients revealed that the increased mechanical loading and strain induced by a higher BMI positively affects bone modeling, density, and geometry (32, 33). Herein, understanding the interaction between obesity and recollapse has become a pressing demand to improve OVCF patient prognosis.

A long-standing perception of IVC as a sign of instability may be a consequence of the fibrocartilaginous membrane surrounding the IVC, which prevents cement diffusion and observes an unsatisfactory bonding between the cement and the surrounding cancellous bone (34). Thus, it is logically a sign of surgical complications including recollapse and residual low back pain (35, 36). Yu et al. systematically reviewed substantial studies and summarily reported a significant therapeutic efficacy in OVCF patients with percutaneous vertebral augmentation, but IVC was responsible for more frequent recollapse and unfavorable radiological parameters compared to controls (37). In a comprehensive perspective exploring the impact of the presence of IVC on PKP efficacy, Li et al. found that patients with IVC were not only associated with a higher incidence of cement leakage but also experienced more severe kyphotic angle rebound and recollapse during follow-up (38). These results suggested that IVC patients might need targeted and individualized long-term health interventions.

As the transition area between the relatively stable thoracic spine and relatively flexible lumbar spine, the thoracolumbar junction concentrates a majority of the vertical load (8, 39, 40). For compression fracture segments, the changed morphology of the vertebral body shifts the center of gravity ventrally and concentrates stresses on the cranial endplate (39). Given the leverage effect exerted by the thoracic vertebrae on the thoracolumbar area, this mechanism was particularly evident in the thoracolumbar junction, and the progressive collapse of compression fracture evolved as an inherent characteristic of this anatomical structure (39). In the present study, of the 40 patients with significant recollapse observed at final follow-up, 27 (67.5%) were diagnosed with fracture at thoracolumbar junction, 11 (27.5%) with fracture at lumbar vertebrae, and 2 (5.0%) with fracture at thoracic vertebrae.

To further investigate the fracture patterns of various anatomical structures on the efficiency of PKP, we analyzed the vulnerable cranial endplate and the anterior cortical wall in patients with OVCFs. Compared to the caudal endplate, the cranial endplate is a more susceptible area to disruption when subjected to excessive vertical loading, as fewer trabeculae are distributed (39, 41). The upper endplate fracture is accompanied by irreparable damage that manifests as progressive collapse and significant local kyphosis at impaired segment after percutaneous vertebral augmentation (39, 42). As the blood supply to the anterior vertebral column is mainly derived from the metaphyseal feeding arteries (43), and for patients with anterior cortical fractures, kyphoplasty is unable to reverse the outcome of ischemic necrosis of the anterior column, a long-term vertebral collapse seems to be foreseeable. We found that at final follow-up, both cranial endplate fractures and anterior cortical fractures were significantly associated with postoperative recollapse, with only the uninvaded segments maintaining favorable vertebral height. Thus, as mentioned above for the risk stratification of fracture patterns, this study accomplished the primary task of evaluating the risk factors, but the management of patients with these complicated OVCFs for a desirable long-term prognosis remained a challenge for spine surgeons to struggle with.

Several studies have elaborated on the important role of cement-related parameters in enhancing vertebral stability (40, 44, 45). Multivariate logistic analysis indicated that separated cement masses and alone upper endplate contact were risk factors for recollapse after PKP. However, no statistical difference in cement injection volume was found between the recollapse and control groups in the current work. The injected cement usually follows the existing fracture cleft diffusely, adequate cement distribution suggests the injured vertebrae are satisfactorily filled and solidly supported, while the sclerotic layer formed by extensively compressed trabeculae limits the cement augmentation. After analyzing 220 patients with OVCFs, He et al. found that cement distribution was positively correlated with vertebral height recovery and kyphotic angle improvement (44). In parallel, the recollapse risk score developed by Yu et al. indicated a higher risk associated with separated cement blocks, which was explained by the weaker cancellous bone not being loaded in tandem and collapsed by normal stress under insufficient cement distribution (46). Meanwhile, whether the bone cement touches the upper and lower endplates has been shown to be a predictor for evaluating recollapse. Zhang et al. described a surgical technique that ensures the injected cement will be in contact with both the upper and lower endplates, and it is effective in reducing the incidence of recollapse (40). Furthermore, the novel X-ray-based scoring algorithm developed by Liu et al. indicated that the ideal cement distribution pattern was exactly that of contacting the upper and lower endplates, and the occurrence of re-collapse of the augmented vertebrae increases by 14.4% as the cement distribution quadrant decreases (47).

In this study, we constructed a superior ML-based classifier with mean AUC of 0.81, maximum AUC of 0.85, and ACC of 0.81. There are certain limitations that undermine the reliability of the study, and further investigations are needed to determine the risk factors for recollapse of OVCFs after PKP. First, although various parameters were analyzed based on clinical experience and literature searches, the definition of recollapse is actually inconsistent across institutions, and recollapse is also associated with multiple factors. Second, this conclusion from a single-center retrospective study may require further validation in a prospective multicenter study. Finally, we only evaluated predictors of recollapse at 1 year postoperatively, and long-term follow-up may identify some new risk factors.

## Conclusion

In summary, the incidence of recollapse in OVCFs was 11.56% during the 1-year follow-up after PKP. Using ML algorithm, we constructed an SVM model with superior predictive performance. Patients with high BMI, low BMD T-scores, presence of IVC, separated cement distribution, cranial endplate or anterior cortical wall violation, cement-contacted upper endplate alone, and thoracolumbar fracture should have enhanced perioperative surveillance and fall prevention education for the target of secondary recollapse prevention.

## Data Availability Statement

The raw data supporting the conclusions of this article will be made available by the authors, without undue reservation.

## Ethics Statement

This study was reviewed and approved by the Ethics Committee of our hospital (Second Hospital of Dalian Medical University, Dalian, People's Republic of China).

## Author Contributions

S-tD and BY completed the study design. S-tD, JZ, and HY performed the study and collected and analyzed the data. S-tD and JZ drafted the manuscript. BY provided the expert consultations and suggestions. S-tD, GH, and CZ conceived the study, participated in its design and coordination, and helped to embellish language. All authors reviewed the final version of the manuscript.

## Funding

This work is supported by the 1+X program for Clinical Competency enhancement–Interdisciplinary Innovation Project, The Second Hospital of Dalian Medical University.

## Conflict of Interest

The authors declare that the research was conducted in the absence of any commercial or financial relationships that could be construed as a potential conflict of interest.

## Publisher's Note

All claims expressed in this article are solely those of the authors and do not necessarily represent those of their affiliated organizations, or those of the publisher, the editors and the reviewers. Any product that may be evaluated in this article, or claim that may be made by its manufacturer, is not guaranteed or endorsed by the publisher.

## Abbreviations

OVCFs: osteoporotic vertebral compression fractures
PKP: percutaneous kyphoplasty
SVM: support vector machine
ML: machine learning
ROC: receiver operating characteristic
ASA: American Society of Anesthesiologists
BMI: body mass index
BMD: bone mineral density
VAS: visual analog scale
VBCR: vertebral body compression rate
AVH: anterior vertebral height
PAH: posterior vertebral height
PAHC: percentage of anterior height compression
IVC: intravertebral vacuum cleft
SD: standard deviation
PROs: patient-reported outcomes
AUC: area under the receiver operating characteristic curve
ACC: accuracy.
